# Mothers' beliefs about infant teething in Enugu, South-east Nigeria: a cross sectional study

**DOI:** 10.1186/1756-0500-4-228

**Published:** 2011-07-01

**Authors:** Gilbert N Adimorah, Agozie C Ubesie, Josephat M Chinawa

**Affiliations:** 1 Department of Paediatrics, Faculty of Medical Sciences, College of Medicine, University of Nigeria, Nigeria

**Keywords:** Teething, Children, associated symptoms, Enugu

## Abstract

**Background:**

Parents and Health Care Workers have traditionally attributed a variety of symptoms to teething in young children. Some of these symptoms may however connote underlying serious medical condition in a child. There is little evidence to support these beliefs despite their implications on management of a symptomatic teething child. This study therefore seeks determine the beliefs and problems mothers associate with teething in Enugu, South-east Nigeria.

**Findings:**

A cross-sectional survey involving sixty mothers presenting at a Children's clinic in Enugu metropolis using questionnaire. More than 90% of the respondents thought that babies can experience medical problems as a result of teething. The commonest medical problems perceived to be associated with teething were fever (71.7%), loose stools (58.3%) and vomiting (35%).

**Conclusion:**

Mothers still associate a variety of symptoms of childhood illnesses to teething and this association is not evidence based and could lead to delayed interventions, increased morbidity and mortality of children. It is important therefore that mothers and health workers caring for young children are educated on the need to seek prompt medical attentions in a symptomatic child.

## Findings

## Background

Teething according to Tasanen cited in Swann [1] has traditionally been the explanation for a variety of symptoms and signs associated with tooth eruption in the young child, both by parents and doctors. A child's first tooth usually appears by 6 months of age, and a complete set of 20 primary or first teeth usually develops by age three [2]. It is important to remember that during this same period of an infant's life, passive immunity due to maternal antibodies wanes and exposure to a wide variety of childhood illnesses can occur [3]. Some of the attributable symptoms such as drooling of saliva and itching gum are trivial, nevertheless significant to the child and parents [4]. Others such as fever, diarrhea and cough may connote underlying serious medical conditions in the child. There is little evidence to support these beliefs despite their implications for prompt diagnosis and management of childhood illnesses [4]. Such uninformed beliefs could cause delays in diagnosing and managing serious childhood illnesses. Delayed diagnosis of underlying serious medical conditions on the other hand, may have far reaching consequences including mortalities from otherwise preventable and treatable diseases. It is important therefore, that parents are knowledgeable about these symptoms, what they can do safely at home, and when they should seek help in a symptomatic child. This study therefore, seek to find the beliefs of mothers from Enugu, South-East Nigeria on childhood teething. It is expected that the findings of the study will help form the basis for health promotional messages aimed at addressing misconception about teething in the zone.

## Methods

### Setting

This study was conducted in a private paediatric clinic in Enugu; the capital of Enugu state, South-east, Nigeria.

### Study design

This was a cross sectional quantitative study using structured questionnaire. The questionnaire was developed based on professional experience, interaction with the mothers and literature review. The advantage of using cross sectional quantitative study was that data could be efficiently obtained in a relatively short time.

### Participants

All women presenting with their babies at the clinic were approached for this study. They belonged to the upper and middle social classes. Consent was obtained from a total of 63 mothers that brought their babies to the clinic. Sixty of them completed the questionnaires giving a response rate of 95.3%. The recruitment of the participants was carried out in February of 2010.

### Materials

The questionnaire used for this study was divided into two sections. The first section on demography contained age of the respondents in the next birthday, highest educational level attained and occupation of respondent and spouse, age of the last child and parity. The second section contained common problems usually attributed to teething and list of possible remedies and interventions that are usually applied by caregivers. A sample of the questionnaire has been included as "Additional file 1".

### Procedures

Data were collected using structured questionnaire. The participants were enrolled consecutively as they presented to the clinic with their children. The purpose of the study and the questionnaire were explained to each participant and consent obtained. Each participant then completed the questionnaire and returned it before leaving the clinic.

### Data Analysis

The data were analyzed using SPSS version 17. Descriptive statistics were used in reporting prevalences. Chi-squared test was used to test for statistical significance of categorical variables. The level of statistical significance used was 0.05 and 95% confidence interval reported.

#### Pilot study

The questionnaire was piloted on 5 mothers who were subsequently excluded from the study. Ambiguous and improperly worded questions were modified after the pilot.

#### Ethical Approval

Ethical approval was obtained from the Health Research and Ethics Committee of the University of Nigeria Teaching Hospital, Ituku-Ozalla, Enugu State, Nigeria.

## Results

### Subjects

A total of 63 mothers participated in this study. They were all literate with the minimum educational qualification being secondary. Sixty of them completed the questionnaire giving a response rate of 95.3%. Participants were aged 24 to 43 years and the age group, 30 to 39 years accounted for the majority (56.7%) of the participants. The distribution of the participants according to their ages is shown in Table 1. Most of the participants and their spouses had either a University first degree or a higher national diploma accounting for 56.7% and 55% of the study population respectively. About half (46.7%) of the respondents had their last baby aged less than one year. The ages of the last child of the participants are presented in Table 2.

**Table 1:** Age groups of the participants

Age group (years)	Frequency (%)
20 to 29	12 (20.0)
30 to 39	34 (56.7)
40 to 49	4 (6.6)
Missing	10 (16.7)

**Total**	**60 (100)**

**Table 2:** Age of the last child of the participants

Age (years)	Frequency (%)
Less than 1	28 (46.7)
1	14 (23.3)
2	7 (11.7)
3 4 5 More than 5	2 (3.3) 4 (6.7) 2 (3.3) 3 (5.0)

**Total**	**60 (100)**

### Teething and Associated Problems

**Figure 1:**
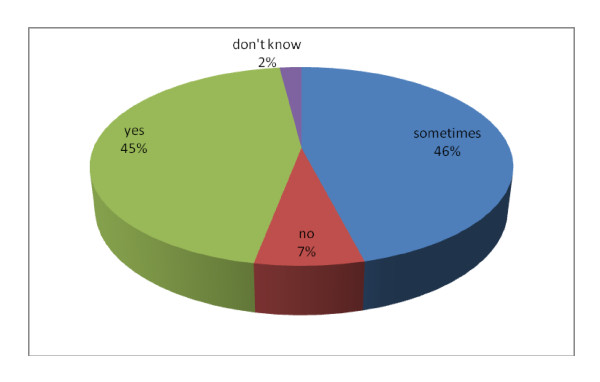
**Participants' responses to whether babies can experience teething problems**.

More than 90% of the respondents thought that babies can experience medical problems as a result of teething; with 45% saying definite yes while 46% said sometimes as shown in Figure 1.

Half of the respondents will worry during teething period while 38.3% and 11.7% do not worry and are not bothered respectively during this period of their child's life. Greater proportion of those that will worry were traders (64.3%) and undergraduates (57.1%) but there was no statistically significant difference among the women that will worry from the various professions (Χ = 22.67; P = 0.65). Similarly, greater proportion of the graduates/higher national diploma holders (58.8%) and post graduates (41.2%) worry about teething in their children compared to those with secondary education, ordinary national diploma or national certificate of education. Among the various age groups, 58.3% of those aged 20 and 29 years worry about teething compared to 52.9% and 25% in the age groups 30 to 39 years and 40 to 49 years respectively. This difference was not statistically significant (F = 1.91; P = 0.16). More of the women currently nursing younger children reported worrying about teething (56.7% among those with a child less than one year, 23.3% among those with children aged one year, 10% respectively for those with children aged two years and more than two years). This difference was statistically significant (F = 4.97; P = 0.01). The commonest medical problems associated with teething were fever (71.7%), loose stools (58.3%) and vomiting (35%) as shown in Table 3. Seventeen mothers (28.3%) reported only one symptom, 38.3% reported between two and three symptoms; only 6.7% reported no symptoms while the rest reported between 4 and 7 symptoms.

**Table 3:** Common medical problems associated with teething

Medical problems	Frequency (%)	95% Confidence Intervals
fever	43 (71.7)	60.6 -83.4
Loose stools	35 (58.3)	45.5 - 70.5
Vomiting poor appetite undue crying	21 (35.0) 18 (30.0) 18 (30.0)	22.9 - 47.1 18.4 - 41.6 18.4 - 41.6
greenish stool abdominal gripes cough	13 (21.7) 9 (15.0) 6 (10.0)	11.5 - 32.5 6.0 - 24.0 2.4 - 17.6

### Management of Teething Associated Problems

The commonest sources of teething information/advice were from respondents' own mothers (26.3%), doctors (22.8%), observing others (15.8%) and friends (12.3%). Only 11.7% of the respondents thought that teething in a child could have effect on the other siblings.

A majority of the respondents (58.4%) consistently apply teething medications during teething whereas 18.3% do not apply anything, 10% do apply sometimes and the rest if need be. Altogether, 49 respondents had applied teething medications. Among th, , the commonest medication used were "teething powder" (powdered asprin and carbonate) in 46.9% of the respondents; varied combination of treatment options (paracetamol, "salt water, aspirin, teething powder, 7 keys, "pican" and "gbomoro"(a herbal concoctions) in 20.3%, paracetamol (18.4%), paracetamol and teething powder alone in 8.2%, 2% each for asprin, 7 keys (herbal concoctions) and salt water as indicated in Table 4. Interestingly, 50% of the respondents believed that nothing will happen to a child even if teething medications are not used during teething while 25% thought that not using teething medications could result in severe illness in a child.

**Table 4:** Management of the teething associated problems by mothers

Treatment given	Frequency (%)
Teething powder Various combinations of treatment options	23 (46.9) 10 (20.4)
Paracetamol	9 (18.4)
Paracetamol and teething powder	4 (8.2)
aspirin salt water 7 keys	1 (2.0) 1 (2.0) 1 (2.0)

Total	49 (100)

The commonest treatments for diarrhea, fever and cough were oral rehydration solution (88.9%), paracetamol (75.9%), and cough syrups (70.2%) respectively. Other management options for diarrhea were herbs (1.9%), "gbomoro) (1.9%), see a doctor (3.7%). Aside paracetamol, other options for managing fever were ibuprofen (10.3%), tepid sponging (1.7%) see a doctor (1.7%) or a combination of management options (10.3%). The women were also likely to use septrin (3.5%), other antibiotics (7.0%), doctor's prescription (7.0%) or a combination of options in treating cough.

## Discussion

This cross sectional survey shows that mothers belonging to the upper and middle class in the Enugu metropolis of South-eastern Nigeria associate common symptoms of childhood illnesses with teething. These beliefs are in spite of lack of supporting medical evidence [5]. The commonest teething associated problem in this study was fever (71.1%) which was similar to the 70% documented by Wake *e al *[5] in their study conducted in Australia. Their study also documented that 36% of their respondents reported loose stools as teething associated, which differs sharply with the 58.3% in this current study. The reason for this difference may not be unconnected with the higher incidence of diarrhea generally in developing countries like Nigeria compared to advanced countries like Australia. In a previous Nigerian study, Uti *et al *[6] found that most of the women (95.2%) in their study associated various symptoms of childhood illness with teething. The two commonest symptoms believed to be associated with teething by their respondents were fever (80.5%) and diarrhea (64%) which agrees with the commonest associated symptoms noted in this current study. Their documented prevalences for the perceived symptoms were however; slightly higher than in this study. This may partly be explained by the larger sample size of their study. They also found that educational status of the mothers did influence perception about teething. This supports our findings that involved only educated upper and middle class mothers. In another study, this time prospective cohort by Wake *et al *[4] involving 21 children of teething age, aged 6 to 24 months in Melbourne, Australia; the authors noted that there are actually no medical symptoms that were significantly associated with teething in a child. They monitored for temperature rise, episodes of diarrhea and appearance of other illnesses during the teething period. Their findings contradicts that of this study and other studies that interviewed parents and health care workers [5] highlighting possible disparity between caregivers' perceptions and observing for teething associated problems in children. Other reviews have refuted this, claiming that teething in children can be associated with problems such as temperature above 38.3°C, drooling, irritability, gum sucking and reduced appetite [7]. Among five groups of health professionals most closely concerned with the health of children (100 maternal and child health nurses, 100 pharmacists, 150 general practitioners, 100 dentists, and 100 paediatricians) in Victoria, Australia the mean number of different symptoms ascribed to teething per group was 2.8 (paediatricians), 4.4 (dentists), 6.5 (general practitioners), 8.4 (pharmacists), and 9.8 (nurses) in their series [5]. Interestingly too, 32 pharmacists and 19 dentists reported that teething may cause fever (> 38°C), compared with seven nurses, 12 general practitioners and two paediatricians. Only nine paediatricians, but 30-50% of each of the other groups, believed that teething predisposes to infections, most commonly colds and ear infections [5]. In every group, most of those who believed that teething cause symptoms ascribed irritability, dribbling or drooling, biting objects, sleep problems, inflamed gums, and red cheeks to teething [5]. Their review showed that paediatricians more than any other professional are less likely to attribute serious symptoms like fever and infections to teething. In another prospective study, increased biting, drooling, gum-rubbing, sucking, irritability, wakefulness, ear-rubbing, facial rash, decreased appetite for solid foods, and mild temperature elevation were all statistically associated with teething while congestion, sleep disturbance, stool looseness, increased stool number, decreased appetite for liquids, cough, rashes other than facial rashes, fever over 39°C, and vomiting were not significantly associated with tooth emergence [8]. It is worth noting in this study, that no symptom occurred in 35% of infants during each teething period, and 20% more often in the teething period than in the non-teething period making it impossible to categorically ascribe any of them to teething [8]. In a systematic review of five articles (two retrospective and three prospective) that focused on the association between teething and systemic symptoms; Tighe and Roe [9] concluded that while a variety of symptoms may occur contemporaneously with teething, there is no pattern of symptoms manifesting in all the studies reviewed that can reliably distinguish teething from any other potential cause of the symptoms. There is therefore, need for cautions in attributing various symptoms to teething alone by parents and health care workers especially symptoms that can be caused by serious childhood illnesses.

About half of our respondents in this study used teething medications compared to 76% in the study by Wake *et al *[7]. The treatment options identified by Wake *et al *[7] in their series were objects to chew, comforting and cuddling, paracetamol, teething gels, gum massage, natural/herbal medicines and sedatives. In contrast, paracetamol/ibuprofen, cough syrups, oral rehydration solution, antibiotics, local remedies, pican, "7 kits" were some of the treatment options utilized by mothers in this study. It can therefore be opined that managing teething associated problems varies from locality to locality and focuses on symptom reliefs [10]. This may portend some danger for the young child especially when the said symptom is not due to teething but a result of other common childhood illness. It is important therefore that mothers are counseled about symptoms of common childhood illness and that a child with fever for instance may be having some other serious medical conditions like severe malaria, meningitis or other infections instead of just dismissing the fever as teething. The use of anti-pyretics should not stop the need to consult a physician if fever persists. The same argument can be made for diarrhea although as much as 88.9% of our respondents correctly said they use oral rehydration solution in managing diarrhea/loose stools.

## Conclusion

Most of the mothers interviewed ascribed symptoms of childhood illnesses to teething. It is important therefore, that women of reproductive age in general, but especially the younger nursing mothers are targeted with health promotion messages that will ensure appropriate and prompt interventions for a symptomatic teething child.

## Competing interests

The authors declare that they have no competing interests.

## Authors' contributions

GNA conceived the study idea, developed the initial questionnaire for the study. He reviewed the draft manuscript making suggestions that informed the final draft. ACU reviewed the data, conducted the data analysis and wrote the first draft manuscript which was shared with the other authors. He collated the corrections made by the other authors. JMC piloted the initial draft questionnaire, collected data and contributed in modifying the initial draft. All the authors participated in the write up, read and approved the final manuscript.

## Supplementary Material

Additional file 1 **Questionnaire**. This is a copy of the questionnaire used in conducting this research. Click here for file
