# Supplementary material for: Mothers' beliefs about infant teething in Enugu, South-east Nigeria: a cross sectional study
Source: BMC Res Notes. 2011 Jul 1;4:228. doi: 10.1186/1756-0500-4-228 (PMC3146856; doi:10.1186/1756-0500-4-228)
Supplement: Additional file 1 — Questionnaire. This is a copy of the questionnaire used in conducting this research. [file 1756-0500-4-228-S1.DOC]

**FAVOUREDCHILD CLINICS**

QUESTIONAIRE ON MOTHERS’ UNDERSTANDING

OF TEETHING AND ASSOCIATED PROBLEMS

**TO ALL MOTHERS**

This questionnaire is designed to help doctors and nurses understand better the problems that mothers may have when their children are teething. This will help us to give better counsel to the mothers, and help the babies better when they are passing through this period of development.

All information given to us will be treated as strictly confidential and will not be made available to any other party.

Your co-operation is highly appreciated. Please help us give your answers as accurately as possible. Thank you.

1. 1)NAME(OPTIONAL)……………………………………………………………………………

2)ADDRESS………………………………………………………………………………………

3)OCCUPATION………………………………………………………………………………..

4)EDUCATIONAL LEVEL………………………………………………………………………..

5)AGE NEXT BIRTDAY……………………………………………………………………………

6)HUSBANDS’OCCUPATION…………………………………………………………………

7)HUSBANDS’ EDUCATIONAL LEVEL………………………………………………………….

8)PARITY…………………………………………………………………………………………..

9)AGE OF LAST CHILD…………………………………………………………………………..

1. Please answer these questions as accurately as you can.
2. Do babies have any problems when their teeth are erupting?

| YES | NO | SOMETIMES | DON’T KNOW |
| --- | --- | --- | --- |
|  |  |  |  |

1. At what age do you expect a baby’s teeth to start erupting?

| 3MTHS | 4MTHS | 5MTHS | 6MTHS | 7MTHS | 8MTHS | B4 1YR |
| --- | --- | --- | --- | --- | --- | --- |
|  |  |  |  |  |  |  |

1. Do you worry about the time your baby’s teeth start to erupt?

| YES | NO | NOT BOTHERED |
| --- | --- | --- |
|  |  |  |

1. What problems do you think are associated with teething in children?

| FEVER | LOSE STOOLS | VOMITING | GREENISH  STOOLS | POOR  APPETITE | COUGH | UNDUE  CRYING | ABD  GRIPES | ANY  OTHER |
| --- | --- | --- | --- | --- | --- | --- | --- | --- |
|  |  |  |  |  |  |  |  |  |

1. Do you apply any medications to your child when you expect him/her to start teething?

| YES | NO | SOMETIMES | IF NEED BE |  |
| --- | --- | --- | --- | --- |
|  |  |  |  |  |

1. What do you usually give your child when he starts teething?

| PARACET | ASPIRIN | TEETHING POWDER | ‘GBOMORO’ | HERBS | SALT WATER | ANY OTHER |
| --- | --- | --- | --- | --- | --- | --- |
|  |  |  |  |  |  |  |

1. Who gave you the information on how to handle teething in babies?

| MOTHER | GRAND MOTHER | FRIENDS | FROM OBSERVATION | NURSE | DOCTOR | PATENT  MED |
| --- | --- | --- | --- | --- | --- | --- |
|  |  |  |  |  |  |  |

1. What do you think will happen if you do not give the baby anything for teething?

| POOR GROWTH | SEVERE  ILLNESS | NOTHING | DEATH | ‘NTA’ | ANY OTHER |
| --- | --- | --- | --- | --- | --- |
|  |  |  |  |  |  |

1. Have you ever seen any child die from teething problems?

| YES | NO | HEARD STORIES | ONLY WARNED |
| --- | --- | --- | --- |
|  |  |  |  |

1. Does teething have any effects on the older children?

| YES | NO | DON’T KNOW |
| --- | --- | --- |
|  |  |  |

1. Which of the teeth erupt first, the upper incisors or the lower ones?

| UPPER | LOWER | NOT SURE |
| --- | --- | --- |
|  |  |  |

1. Does it matter which of them erupts first?

| YES | NO | DON’T KNOW |
| --- | --- | --- |
|  |  |  |

1. If yes,why does it matter?

|  |
| --- |

1. If a baby develops teething problems who will you prefer to take him to first?

| DOCTOR | PAT. MEDICINE | NURSE | HERBALIST | CAN HANDLE | RELIABLE  NEIGHBOUR | GRAND  MOTHER | NOBODY |
| --- | --- | --- | --- | --- | --- | --- | --- |
|  |  |  |  |  |  |  |  |

1. If the teething problem is diarrhoea, what remedy will you give?

| HERBS | DIAPEC | ORS | JUST WAIT | GBOMORO | BITTERS | ANY OTHER |
| --- | --- | --- | --- | --- | --- | --- |
|  |  |  |  |  |  |  |

1. If the teething problem is fever, what remedy will you give?

| PARACETAMOL | HERBS | TEETHING  POWDER | TEPID  SPONGING | IBUPROFEN | NOTHING |
| --- | --- | --- | --- | --- | --- |
|  |  |  |  |  |  |

1. If the teething problem is cough, what remedy will you give?

| COUGH SYR | SEPTRIN | OTHER  ANTIBIOTICS | HERBS | ACTIFED | ANY  OTHER |
| --- | --- | --- | --- | --- | --- |
|  |  |  |  |  |  |

1. Is teething related to abdominal gripes (pains) in children?

| YES | NO | DON’T KNOW |
| --- | --- | --- |
|  |  |  |

1. What remedy do you use if you think they are related?

| GRIPE WATER | SALT WATER | ANTACIDS | HERBS | ANY OTHER | DON’T KNOW |
| --- | --- | --- | --- | --- | --- |
|  |  |  |  |  |  |

THANK YOU
